# Editorial: Threats and strategies of nutritional metabolic disorders and poisoning diseases in ruminants

**DOI:** 10.3389/fvets.2026.1817108

**Published:** 2026-03-27

**Authors:** Baochen Fang, Zhifeng Chen, Mengjie Li, Pu Wang, Hui Chen, Lihui Guo, Zhicheng Peng

**Affiliations:** 1 Department of Plant Sciences, North Dakota State University, Fargo, ND, United States; 2 Department of Data Science and AI (DSAI), Monash University, Clayton, VIC, Australia; 3 Institute of Research and Clinical Innovations, Neusoft Medical Systems Co., Ltd., Hangzhou, China; 4 Department of Chemistry and Biochemistry, Texas Tech University, Lubbock, TX, United States; 5 Yunnan Provincial Key Laboratory of Animal Nutrition and Feed, Faculty of Animal Science and Technology, Yunnan Agricultural University, Kunming, China; 6 Zhejiang Jinda Kang Animal Health Products Co., Ltd., Jinhua, China; 7 Ragon Institute of Mass General Brigham, Massachusetts Institute of Technology (MIT), and Harvard University, Cambridge, MA, United States; 8 Institute of Biotherapy, Henan Academy of Innovations in Medical Science, Zhengzhou, China; 9 Department of Experimental Immunology, Amsterdam Infection and Immunity Center, Amsterdam University Medical Centers (UMC), Academic Medical Center (AMC), University of Amsterdam, Amsterdam, Netherlands; 10 Department of Cancer Biology, Abramson Family Cancer Research Institute, Perelman School of Medicine at the University of Pennsylvania, Philadelphia, PA, United States

**Keywords:** animal health, immune dysfunction, infectious diseases, nutritional metabolic disorders, ruminants

Ruminants, including cattle, sheep, goats, deer, giraffes, antelopes, and bison, play a central role in global livestock production, providing essential products such as milk, meat, leather, wool, and medicinal resources (1). However, their unique digestive physiology, metabolic characteristics, and patterns of energy utilization render them particularly susceptible to physiological disturbances, particularly during the transition period spanning late gestation to early lactation (1, 2). During this period, animals undergo significant physiological and nutritional stress due to the abrupt transition from low- to high-energy diets, accompanied by dramatic changes in hormones (3, 4). Intense metabolic load, imbalances in energy, protein, and minerals, or exposure to dietary toxins can disrupt rumen ecology, impair hepatic lipid and glucose metabolism, and compromise systemic immune function (5, 6). Collectively, these perturbations increase susceptibility to metabolic, inflammatory, and infectious disorders and are commonly associated with multi-organ dysfunction involving the liver, reproductive tract, gastrointestinal system, and immune tissues (7–9).

The consequences are profound. Affected animals show reduced performance, altered milk composition and quality, increased morbidity and mortality, and considerable economic losses (10, 11). Nutritional metabolic disorders and poisoning-related conditions remain major constraints on the animal health, productivity, and welfare of ruminant livestock worldwide (12). Their multifactorial etiology demands integrated strategies

that combine precision nutrition, feed safety, rumen health management, and real-time monitoring. Understanding the pathogenesis and mitigation of these disorders is therefore essential not only for preventing and treating nutrition-related metabolic diseases but also for ensuring sustainable livestock production and global food security. Strengthening preventive programs and adopting emerging technologies are crucial steps toward improving animal welfare, productivity, and the sustainability of ruminant industry.

This Research Topic aims to provide a comprehensive and integrative perspective on nutritional metabolic disorders, poisoning diseases, immune dysfunction, and infection-associated complications in ruminants. Specifically, it aims to investigate the etiology and pathophysiology of these conditions, understand their interconnected mechanisms, and assess their broader impacts on animal health. Key objectives include enhancing diagnostic capabilities, clarifying disease progression, optimizing management and treatment strategies, and developing effective preventive measures. Additionally, the Research Topic explores technological advances in animal health and their potential application in addressing these challenges. The collection of studies presented here offers timely insights into the multifactorial nature of these disorders and highlights emerging nutritional, microbial, and management-based strategies for their mitigation. The Research Topic encompasses mechanistic investigations, nutritional interventions, diagnostic advancements, and comprehensive reviews, providing a multidimensional perspective on disease prevention and metabolic regulation in ruminants.

One of the central themes across this Research Topic is the application of functional feed additives to enhance metabolic stability and immune competence. Several studies demonstrate that targeted nutritional supplementation improves growth performance while modulating rumen or gut ecosystems. For example, Xue et al. reported that supplementation with exogenous non-starch polysaccharides improved average daily gain and feed efficiency in fattening sheep, with 0.3% inclusion yielding the most favorable results. The supplementation also enhanced rumen fermentation, increased immunoglobulin concentrations, and reduced viral abundance in the rumen microbiome, suggesting potential mitigation of inflammatory challenges associated with high-concentrate feeding. Yeast-derived products have also received considerable attention. Chen, Lui et al. demonstrated that yeast culture and β-glucan supplementation improved growth performance, antioxidant status, and immune function in Mongolian ram lambs, alongside favorable shifts in metabolic hormones. Similarly, Li, Yang et al. reported that yeast culture improved antioxidant enzyme activity, immunoglobulin concentrations, and intestinal microbial diversity in Simmental beef cattle. In preweaning calves, yeast culture reduced diarrhea incidence and promoted beneficial microbial taxa, underscoring its value across developmental stages. Complementing these findings, Liu et al. observed enhanced feed efficiency, antioxidant capacity, and economic returns in Hu sheep receiving compound yeast culture. Beyond yeast products, Cheng, Liu et al. demonstrated that niacin supplementation in sheep fed high-concentrate diets improved ruminal pH, reduced lactate accumulation, and reshaped microbial and metabolic pathways linked to the tricarboxylic acid cycle. Yu et al. similarly showed that rumen-protected glucose supplementation in early-lactation cows elevated blood glucose, reduced Non-Esterified Fatty Acids (NEFAs) and β-Hydroxybutyric acid (BHBA) concentrations, and improved milk yield and composition, indicating improved energy balance. Collectively, these studies highlight nutritional modulation as a practical and scalable approach to preventing metabolic disturbances in ruminants.

Several studies addressed metabolic diseases, like ketosis-related disorders, associated with negative energy balance, particularly during the transition period. Guo et al. provided evidence that postpartum insulin supplementation in obese dairy cows reduced NEFAs and BHBA concentrations and lowered the incidence of type II ketosis. Mechanistic analysis revealed upregulation of Sterol Regulatory Element Binding Transcription Factor 1 (SREBF1) and downstream lipogenic pathways, offering insight into improved insulin sensitivity and adipose metabolism. Pregnancy toxemia, another critical metabolic disorder, was explored by Chen, Wang et al., who observed marked alterations in rumen microbial composition in affected ewes. Reductions in fiber-degrading and short-chain fatty acid-producing genera were closely associated with hypoglycemia and elevated BHBA, highlighting the tight linkage between rumen ecology and systemic energy metabolism. From a developmental perspective, Ramos dos Santos et al. showed that maternal metabolic status and seasonality significantly influenced calf immunity and health outcomes. High maternal condition scores increased diarrhea risk in offspring, while lower maternal condition was associated with stronger innate immune responses. These findings emphasize the importance of prenatal metabolic management in disease prevention.

Rumen acidosis remains a central nutritional disorder in high-production systems. Burgos et al. revisited the role of D-lactate, proposing that this metabolite actively modulates inflammatory responses involving synoviocytes and immune cells, contributing to systemic complications. Complementing this, Mao and Wang highlighted the underappreciated role of rumen foam formation and CO_2_ accumulation in acidosis development under high-concentrate feeding. At the cellular level, Xiao et al. demonstrated that κ-carrageenan oligosaccharides protected ovine rumen epithelial cells from muramyl dipeptide-induced inflammatory injury by suppressing the Nucleotide-binding oligomerization domain 2 (NOD_2_/Nuclear Factor kappa-light-chain-enhancer of activated B cells (NF-κB) pathway, reducing oxidative stress, and improving barrier integrity. This work provides mechanistic support for nutritional modulation of epithelial inflammation during subacute ruminal acidosis.

Microbial homeostasis in ruminants is a critical and indispensable topic, as illustrated by Cheng, Zheng et al., who investigated the etiology of floppy kid syndrome. Biochemical and metagenomic analyses revealed profound gastric and intestinal dysbiosis, with expansion of pathogenic bacteria and loss of beneficial Lactobacillus species. Resulting lactic acidosis and hypoglycemia were identified as key drivers of clinical disease, highlighting microbiota-targeted preventive strategies. Similarly, Ding et al. demonstrated that a combination of a Chinese herbal formula and Microcin J25 effectively inhibited multidrug-resistant *E. coli* and *Salmonella*, reducing diarrhea incidence in calves and providing a promising antibiotic-alternative approach.

Advances in diagnostic and methodological approaches are increasingly vital compared to mechanistic studies, and precise monitoring of metabolic status is critical for early intervention. dos Reis et al. evaluated a human-designed continuous glucose monitoring system in sheep, reporting strong correlations with conventional assays, although inter-animal variability and calibration issues require further validation. This study points toward the future integration of precision livestock monitoring technologies. Finally, this Research Topic further highlights studies that enhance ruminant production performance through improved feeding management and innovative feeding strategies. Zhou et al. assessed the combined use of feed-grade urea and urease inhibitor in beef cattle diets. While growth performance remained unaffected, improved nutrient digestibility and controlled ruminal ammonia dynamics suggest that properly managed non-protein nitrogen sources can reduce feed costs without compromising animal health, provided risks of alkalosis are carefully monitored.

Collectively, the studies in this Research Topic underscore the multifactorial nature of nutritional and metabolic disorders in ruminants, encompassing dietary composition, microbial ecology, endocrine regulation, environmental stress, and developmental programming. Emerging evidence supports precision nutritional strategies, including enzyme preparations, yeast cultures, protected energy sources, and targeted bioactives, as effective tools to enhance metabolic resilience and immune competence. Mechanistic investigations into rumen microbiota, inflammatory mediators, and cellular signaling pathways are reshaping our understanding of disease pathogenesis. Future research should prioritize integrative multi-omics approaches, longitudinal monitoring, and field-validated interventions to translate these findings into practical solutions for herd management and sustainable ruminant production.

## References

[1] Rout PK Behera BK Rout P Behera B. Sustainability in ruminant livestock. Manag Market. (2021) 2021:33–76. doi: 10.1007/978-981-33-4343-6_3

[2] Tufarelli V Puvača N Glamočić D Pugliese G Colonna MA. The most important metabolic diseases in dairy cattle during the transition period. Animals. (2024) 14:816. doi: 10.3390/ani14050816 38473200 PMC10930595

[3] Pascottini OB Leroy JL Opsomer G. Metabolic stress in the transition period of dairy cows: focusing on the prepartum period. Animals. (2020) 10:1419. doi: 10.3390/ani10081419 32823892 PMC7460369

[4] Roche JR Bell AW Overton TR Loor JJ. Nutritional management of the transition cow in the 21st century-a paradigm shift in thinking. Anim Prod Sci. (2013) 53:1000–23. doi: 10.1071/AN12293

[5] Fu Y He Y Xiang K Zhao C He Z Qiu M . The role of rumen microbiota and its metabolites in subacute ruminal acidosis (SARA)-induced inflammatory diseases of ruminants. Microorganisms. (2022) 10:1495. doi: 10.3390/microorganisms10081495 35893553 PMC9332062

[6] Gross JJ. Hepatic lipidosis in ruminants. Vet Clin: Food Anim Pract. (2023) 39:371–83. doi: 10.1016/j.cvfa.2023.02.007 37032295

[7] Aleri JW Hine BC Pyman MF Mansell PD Wales WJ Mallard B . Periparturient immunosuppression and strategies to improve dairy cow health during the periparturient period. Res Vet Sci. (2016) 108:8–17. doi: 10.1016/j.rvsc.2016.07.007 27663364

[8] Plaizier JC Mesgaran MD Derakhshani H Golder H Khafipour E Kleen JL . Enhancing gastrointestinal health in dairy cows. Animal. (2018) 12:s399–418. doi: 10.1017/S1751731118001921 30139397

[9] LeBlanc SJ. Interactions of metabolism, inflammation, and reproductive tract health in the postpartum period in dairy cattle. Reprod Domest Anim. (2012) 47:18–30. doi: 10.1111/j.1439-0531.2012.02109.x 22913557

[10] Bednarski M Kupczyński R. Factors affecting milk productivity, milk quality and dairy cow health. Animals. (2024) 14:3707. doi: 10.3390/ani14243707 39765611 PMC11672682

[11] Kerslake JI Amer PR O'neill PL Wong SL Roche JR Phyn CV. Economic costs of recorded reasons for cow mortality and culling in a pasture-based dairy industry. J Dairy Sci. (2018) 101:1795–803. doi: 10.3168/jds.2017-13124 29248220

[12] Rivero MJ Lee MR A. perspective on animal welfare of grazing ruminants and its relationship with sustainability. Anim Prod Sci. (2022) 62:1739–48. doi: 10.1071/AN21516

